# Non-canonical microproteins as targets for shared-antigen cancer immunotherapy

**DOI:** 10.3389/fimmu.2026.1984200

**Published:** 2026-10-29

**Authors:** Ivo Fierro-Monti

**Affiliations:** 1 European Molecular Biology Laboratory’s European Bioinformatics Institute (EMBL-EBI), Hinxton, Cambridgeshire, United Kingdom; 2 Biozentrum, University of Basel, Basel, Switzerland

**Keywords:** cancer immunotherapy, immunopeptidomics, microproteins, non-canonical open reading frames (ncORFs), tumour antigens

## Abstract

Recent research reveals that microproteins encoded by non-canonical open reading frames (ncORFs) substantially enrich the cancer immunopeptidome, driven by oncogenic stress, essential cellular functions, immune tolerance mechanisms, and antigen-processing biases. These microproteins, often critical for tumour survival, produce peptides presented on tumour cell surfaces, offering shared antigen targets for immunotherapy, particularly in tumours with low mutational burden. Here we integrate multi-level evidence demonstrating how translational reprogramming under stress, essentiality constraints limiting antigen loss, and selective antigen processing converge to promote ncORF-derived peptide presentation. While emerging data suggest immunogenicity of some microproteins, clinical validation remains pending. Advancing therapeutic applications requires development of specialised computational predictors, rigorous immunopeptidomics workflows, and prospective thymic peptide profiling to ensure efficacy and safety. This framework highlights ncORF-derived microproteins as promising yet complex targets, expanding the landscape of cancer immunotherapy beyond canonical neoantigens.

## 1 Introduction

Recent discoveries have revealed that regions of the genome once dismissed as non-coding encode microproteins, many of which play important roles in cancer biology and may serve as novel immunotherapy targets. This phenomenon has been demonstrated at scale by the TransCODE consortium; for example, Deutsch et al. reported that across 95,520 mass spectrometry (MS) experiments, approximately 25% of 7,264 catalogued non-canonical open reading frames (ncORFs) yielded detectable peptides (evidence Tier T1; see **Box 1**) (1). An independent database of unannotated ORFs built from Ribo-Seq and MS corroborated widespread ncORF translation (T1,T2) (2), and Chen et al. demonstrated pervasive functional translation across the human genome (T1) (3). These are not background artefacts: CRISPR screening of 553 non-canonical regions identified 57 loci whose disruption induced viability defects in cancer cells (T1,T3), including GREP1, preferentially essential in breast cancer (4), and OLMALINC, a long non-coding RNA (lncRNA) whose encoded protein product has not yet been fully characterised at the molecular level but whose locus is pan-essential across 85% of 485 tested cell lines as determined by CRISPR dropout (T1, by functional proxy) (1), note that CRISPR dropout alone establishes locus essentiality but not the identity or mechanism of the relevant protein product. Schlesinger et al. found narrower effect sizes and limited overlap with prior screens (T1,T3) (5), highlighting the variability between CRISPR datasets. Nevertheless, the cumulative evidence supports productive, biologically relevant ncORF translation. The repertoire of peptides presented on the tumour surface from this previously uncharacterised proteome is notable. Most ncORF-derived peptides in the TransCODE dataset were detected via HLA immunopeptidomics rather than conventional tryptic proteomics (T2) (1), a discovery mode compatible with transient synthesis and rapid protein turnover. Tumour-specific MHC-I peptides derived from ncORFs, some carrying somatic mutations, were isolated directly from cancer specimens (T2) (2). Within the reported 33 lncRNA-encoded antigens shared across more than 10% of liver cancer patients, 17 had Ribo-Seq translation support and 13 had direct HLA peptide elution evidence (6); RNA prevalence, translation evidence, and HLA presentation are non-overlapping evidence categories applicable to distinct subsets of this group. What appears on the cell surface is actively selected by the antigen-processing machinery.

Box 1 **Evidence tier classification.**

Evidence in this research field can be categorised into Tier 1 (translation), Tier 2 (HLA presentation), and Tier 3 (immunogenicity/function). These tiers are distinct and non-interchangeable; satisfying a higher tier requires independent evidence beyond that supporting the lower tier.

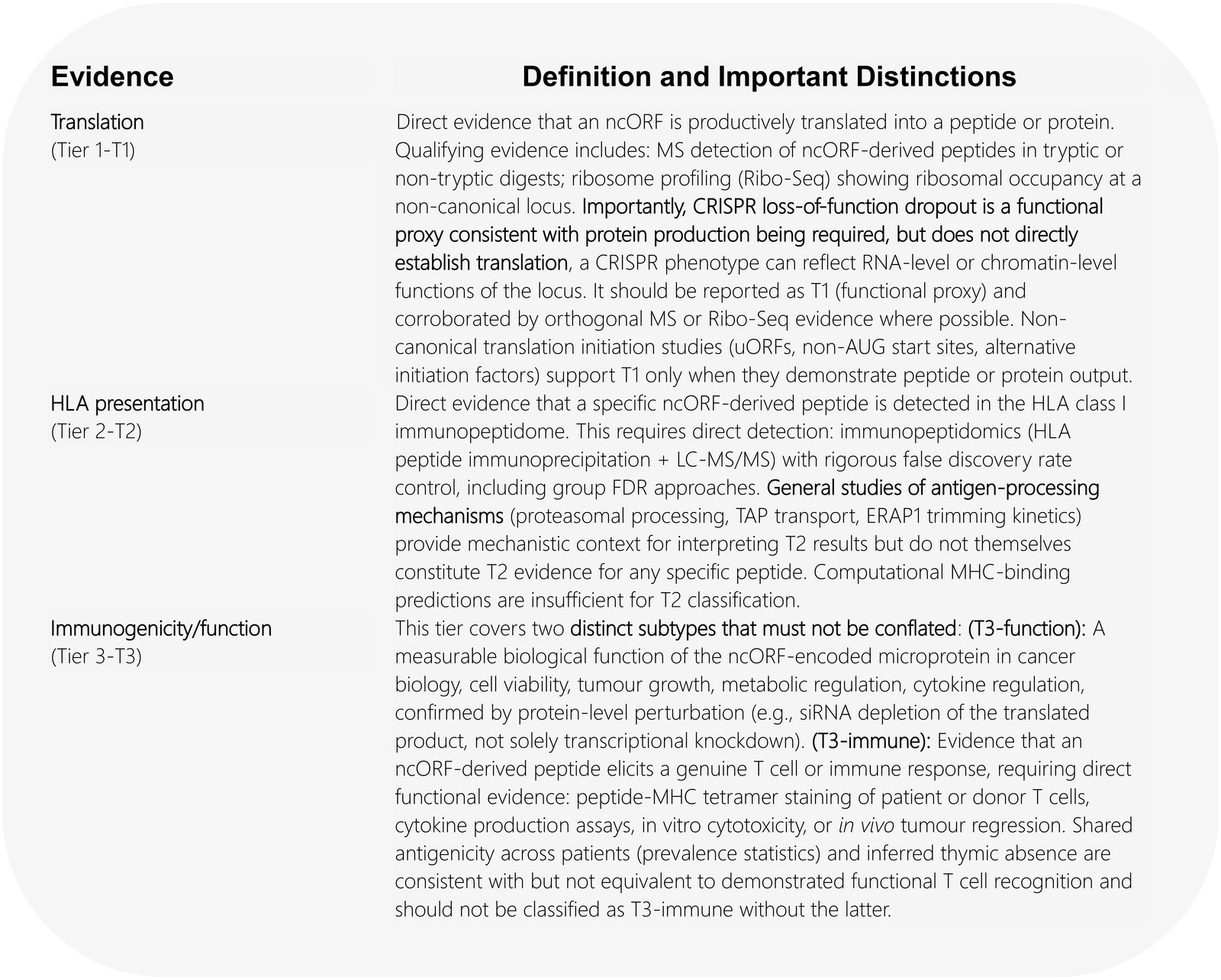

The functional and immunological incentives of that selection are documented across tumour types. Cancer-associated microprotein CASIMO1 (also designated SMIM22) modulates lipid droplet formation in breast cancer cell lines (T3-function) (8) by interacting physically with squalene epoxidase (SQLE). The evidence comes from siRNA-mediated depletion and overexpression experiments rather than from a characterised somatic variant; the functional readouts are proliferation and lipid metabolism, not antigen presentation and T cell recognition. GREP1 regulates the oncogenic cytokine GDF15 (T1,T3) (4). In pancreatic cancer, a disease with few somatic mutations and a poor immunotherapy record, ncORF-derived peptides provide cryptic T cell targets beyond conventional candidates (T2,T3) (9), which may be particularly relevant in settings where canonical approaches fail. Because microproteins are short, any single amino acid substitution affects a larger fraction of the protein’s sequence than in canonical proteins; for example, a single change alters 2% of a 50-residue microprotein versus 0.2% of a 500-residue protein. Studies of the 56-residue Gβ1 domain have shown that 32% of single mutations are destabilising (10); large-scale analysis of *de novo* designed small proteins yields similarly high destabilisation rates (11). However, this observation derives from a well-folded, thermodynamically stable protein domain, and cannot be directly extrapolated to the broader heterogeneous population of human microproteins, many of which are intrinsically disordered and thus have fundamentally different sequence–stability–function relationships. In disordered microproteins, the effects of somatic mutations on protein stability, proteasome production of peptides, and HLA binding affinity remain largely unmapped and must be treated separately empirically. The arithmetic argument above is therefore a motivation for investigation rather than established evidence of increased immunogenic vulnerability. Variant-effect models specifically trained on microprotein deep mutational scanning data, rather than tools calibrated for large, evolutionarily conserved canonical proteins, will be required to resolve this empirically. Thirty-three lncRNA loci occur in more than 10% of liver cancer patients by RNA prevalence (T1 partial), of which 17 have Ribo-Seq translation support and 13 have direct HLA peptide elution confirmation (T2) (6); the subset with T2 evidence provides a foundation sufficient for shared-antigen vaccine design and could provide options for patients with low-mutation tumours where personalised approaches have failed.

Realising this potential requires tools calibrated to the task. Computational predictors such as SIFT (12), PolyPhen-2 (13), and AlphaMissense (14) were trained on large, evolutionarily conserved canonical proteins and underperform on short, disordered peptides, which have different calibration requirements and functional endpoints. Immunopeptidomics databases must incorporate ncORF sequences with rigorous false discovery rate control (T2 methodological validation) (2, 15–17), as inadequate control can lead to sequence ambiguity and subsequent errors in peptide attribution. Focusing exclusively on mutation-derived antigens risks missing a substantial portion of the potential target landscape. Therefore, variant-effect models specifically trained on microprotein deep mutational scanning data are required, rather than relying on conventional tools developed for canonical proteins.

Beyond methodological considerations, fundamental biological questions remain regarding the factors that shape the immunopeptidome. While the genome provides the blueprint, elements such as translational biochemistry, cellular stress states, evolutionary immune history, and selective peptide processing all play critical roles. Notably, ncORF-derived peptides appear at levels that cannot be explained by transcript abundance alone. To account for this phenomenon, four integrated hypotheses are proposed, each representing a distinct causal pathway that may explain the prominence of these peptides.

## 2 A bidirectional framework for ncORF immunogenicity

The prominence of ncORF peptides in the cancer immunopeptidome may be influenced by four mechanistically distinct forces that operate at different levels of biological organisation (see **Figure 1**; **Box 2**). The term ‘bidirectional causation’ in the title of **Figure 1** refers to two complementary causal directions that should be held distinct: upward causation, running from molecular and cellular processes, ncORF translation, proteasomal processing, antigen presentation, to the content of the tumour immunopeptidome; and downward causation, in which the organism’s evolutionary and developmental immune history, specifically, the shape of the T cell repertoire as sculpted by prior thymic selection events, constrains which presented peptides can be immunogenic, independently of their cellular abundance. These are complementary determinants rather than a reinforcing causal loop, and the latter does not directly determine the quantity of peptide displayed by the tumour: T cell repertoires are shaped by thymic selection, while antigen presentation is controlled by cellular processes described in H1, H2, and H4. The four hypotheses therefore address two analytically separable questions: what determines ncORF peptide abundance on the tumour surface (H1, H2, H4), and what determines whether those peptides are immunogenic (H3, and the T cell repertoire shaped by prior selection).

**Figure 1:**
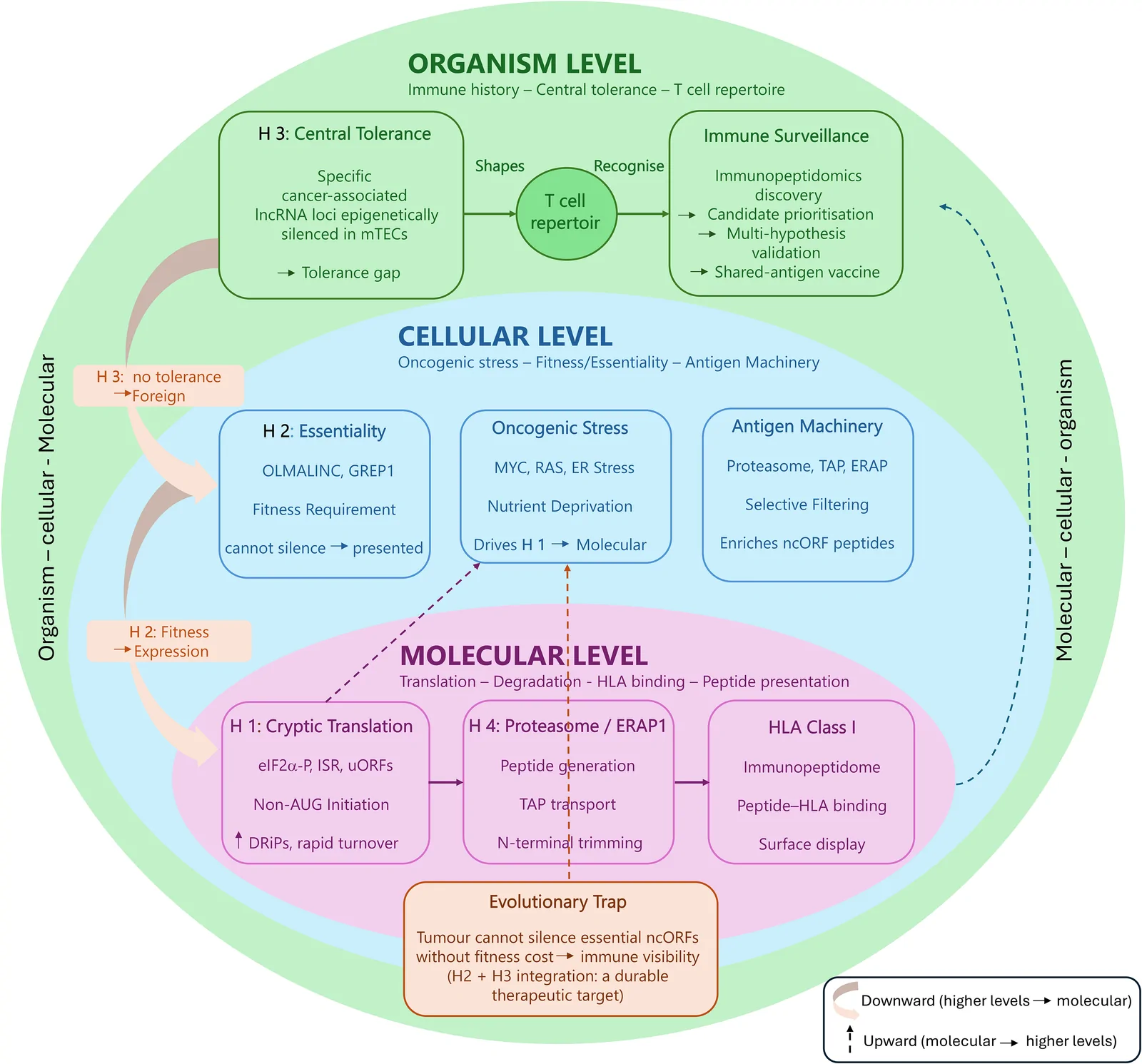
ncORF-derived microproteins as cancer immunotherapy antigens: four integrated hypotheses and bidirectional causation across levels of biological organisation. The diagram organises the four hypotheses (H1–H4) across three nested levels of biological organisation. Organism level (green): H3 represents the central tolerance hypothesis; AIRE-controlled thymic selection shapes the T cell repertoire. For the specific subset of cancer-associated lncRNA loci epigenetically silenced in medullary thymic epithelial cells (mTECs) but derepressed in tumour cells, reactive T cell clones may be retained, enabling immune surveillance upon cancer-driven antigen presentation. This constitutes downward causation: the organism’s developmental immune history constrains which molecularly presented antigens can be immunogenic. Cellular level (blue): H2 (Essentiality, OLMALINC, GREP1; fitness requirement constrains protein silencing as an escape route) and Oncogenic Stress (MYC, RAS, ER stress, nutrient deprivation; drives H1 at the molecular level via eIF2α phosphorylation) and Antigen Machinery (Proteasome, TAP, ERAP1; upstream processing steps that may selectively enrich ncORF-derived peptides; H4). Molecular level (pink): H1 (Cryptic Translation, eIF2α phosphorylation, ISR, uORFs, non-AUG initiation, elevated DRiPs and rapid turnover) feeds into Proteasome/ERAP1 processing (peptide generation, TAP transport, N-terminal trimming; the level at which H4 enrichment is proposed to operate) and HLA Class I surface display (immunopeptidome; peptide–HLA binding is source-agnostic at this step). Upward causation (dashed right arrow): molecular events, translation, processing, surface presentation drive immunopeptidome content independently of the organism’s immune history. Evolutionary Trap (orange box): the H2+H3 integration represents a durable therapeutic opportunity; tumours cannot silence essential ncORFs without proliferative fitness cost. At the same time, the retained T cell repertoire may recognise the resulting antigens as foreign. Hypotheses and evidence tiers are defined in **Boxes 1** and **2**. AIRE, autoimmune regulator; DRiPs, defective ribosomal products; ERAP1, endoplasmic reticulum aminopeptidase 1; ISR, integrated stress response; lncRNA, long non-coding RNA; mTEC, medullary thymic epithelial cell; MHC-I, major histocompatibility complex class I; ncORF, non-canonical open reading frame; TAP, transporter associated with antigen processing; uORF, upstream open reading frame.

Box 2 **The four hypotheses of ncORF immunogenicity, with discriminating experimental predictions.**

Hypothesis 1 (H1): Oncogenic stress drives cryptic translational reprogramming. Stress kinases (PERK, PKR, GCN2, HRI) phosphorylate eIF2α, weakening canonical cap-dependent translation and permitting ribosomal scanning to uORFs, non-AUG sites, and cryptic lncRNA products. The primary amplification is translational, not transcriptional: ncORF peptides can be produced from pre-existing transcripts without any change in transcript abundance. MYC/RAS-driven transcriptional noise is a secondary, contributing mechanism that expands the pool of ncORF-encoding transcripts, but it is not a requirement for ncORF presentation under H1. The resulting microproteins are rapidly degraded and fed into the HLA class I pathway via the DRiP route. Discriminating experimental test: Pharmacological modulation of the ISR (e.g., ISRIB-mediated eIF2B activation to suppress ISR, or PERK inhibitor treatment) combined with matched immunopeptidomics at stable HLA surface levels. A result falsifying H1 as a presentation mechanism: ISR inhibition producing no change in ncORF peptide detection relative to canonical controls, after normalising for total HLA surface expression, cell viability, and global translation rates, the last being critical to exclude confounding by general translation suppression rather than ncORF-specific effects.

Hypothesis 2 (H2): Essentiality creates a conditional presentation liability, not a guarantee. CRISPR screens identify dozens of ncORFs whose disruption impairs cancer cell survival; these proteins must be translated, and their constitutive turnover generates peptides that can enter the HLA class I pathway. Crucially, essentiality constrains source-protein silencing as an immune evasion strategy by imposing a proliferative fitness cost, but it does not guarantee presentation of any specific epitope. Translation of an essential ncORF increases the probability that some peptides will be presented, but as established in the field (7), only a small fraction of detectable proteins contribute to the immunopeptidome. Furthermore, immune evasion through HLA class I downregulation would remove the peptide-HLA target despite continued protein expression, a mechanism that H2's essentiality constraint does not impede. H2 should therefore be understood as constraining one evasion route (protein silencing), not as preventing all evasion routes. Discriminating experimental test: The cleaner test of the essentiality constraint is longitudinal: under sustained antigen-specific immune pressure in vivo, through vaccination or adoptive T cell transfer in isogenic tumour models with intact HLA expression, essential ncORF antigens should exhibit lower rates of antigen-loss escape than non-essential ncORF comparators. ORF-specific versus transcriptional perturbations may additionally be used to evaluate whether antigen presentation changes are translation-dependent, but no single expected direction between these perturbation modes can be specified a priori, as transcript depletion reduces antigen source while accelerated protein degradation can increase peptide substrate availability. A result falsifying H2 as an independent constraint: Essential ncORF antigens exhibiting the same rate of antigen-loss escape as non-essential comparators under sustained immune selection pressure in isogenic tumour models with intact HLA expression.

Hypothesis 3 (H3): Specific cancer-associated lncRNA antigens may escape thymic tolerance, but this does not apply to ncORFs as a class. Existing datasets including the HLA Ligand Atlas (40) and the Peptides for Cancer Immunotherapy (PCI) database (41) demonstrate that ncORF-derived peptides are detectable in healthy thymus and other normal tissues; ncORFs are not generically absent from the thymic peptidome. H3 applies specifically to the subset of cancer-associated lncRNA loci that are epigenetically silenced in medullary thymic epithelial cells (mTECs) under normal developmental conditions but are derepressed in tumour cells. For this subset, T cell clones reactive to the encoded peptides may be retained in the peripheral repertoire. Two critical distinctions must be maintained: (a) absence of thymic mRNA expression in bulk RNA-seq data is not equivalent to absence of peptide-HLA presentation on mTECs, which can present antigens below RNA-seq detection limits; and (b) thymic absence does not exclude peripheral off-tumour injury; the safety question also requires assessing expression and presentation in stressed normal tissues (see H1 and Section 4.3). The prediction that vaccines against H3 candidates would require lower antigen doses than canonical neoantigens remains unsupported by quantitative data and should not be asserted. Discriminating experimental test: Prospective mTEC-specific immunopeptidomics from healthy donors using targeted LC-MS/MS methods, specifically parallel reaction monitoring (PRM) with synthetic isotope-labelled peptide standards for the specific antigen candidates, combined with precursor-frequency assays for reactive T cell clones. Data-dependent acquisition alone is insufficient for this question. A result falsifying H3: Detection of the specific candidate peptide on healthy mTECs at biologically relevant levels, and/or absence of reactive T cell clones at elevated precursor frequency in healthy donors.

Hypothesis 4 (H4): Upstream antigen-processing steps may favour ncORF-derived peptide production, but HLA binding itself is source-agnostic. Short, disordered ncORF proteins with rapid turnover kinetics may be kinetically favoured substrates for DRiP-mediated proteasomal processing and ERAP1 trimming, generating HLA-compatible peptide lengths at higher frequency than longer, folded canonical proteins. This enrichment operates upstream of HLA loading: once a peptide has been generated and imported into the ER, the biochemistry of peptide-HLA association is determined entirely by the peptide's sequence and anchor residues and is identical for canonical and non-canonical sources; ncORF-derived peptides are not inherently 'better binders'. H4 therefore does not predict intrinsic HLA-binding affinity and claims of more favourable anchor-residue profiles for ncORF peptides as a class are not supported by current evidence. Additionally, a key confound must be addressed: immunopeptidomics is inherently more sensitive than tryptic proteomics for short peptides, creating a potential discovery bias that must be distinguished from true biological enrichment. Discriminating experimental test: Allele-controlled comparison of ncORF-derived versus canonical peptide length distributions and HLA occupancy in the immunopeptidome, normalised against source protein turnover rates and matched for HLA allele; ERAP1 knockout or inhibition with allele-controlled motif and length analysis of the ncORF-specific fraction. A result falsifying H4 as a processing-level mechanism: No difference in length distribution or HLA occupancy between ncORF-derived and size-matched canonical peptides after correction for source protein abundance, turnover rate, and detection opportunity.

To illustrate how the multi-tier framework in **Box 1** applies in practice, **Table 1** summarises the current state of evidence for the microproteins and ncORF antigen sets discussed in this article, with denominators for the prevalence claims explicitly stated.

**Table 1:** Candidate-level evidence summary.

Antigen/locus	Genomic origin	T1: translation	T2: endogenous HLA presentation	T3-function: protein-level biology	T3-immune: functional T cell recognition	Cancer type	HLA allele(s)	Reference
GREP1	ncORF	Yes: tryptic MS + Ribo-Seq	Not reported	Yes: essential in breast cancer; regulates GDF15	Not reported	Breast	Not determined	(4)
OLMALINC	lncRNA	Functional proxy only (CRISPR dropout); direct protein detection not confirmed	Not reported	Yes: pan-essential in 85% of 485 cell lines; protein product not characterised	Not reported	Pan-cancer	Not determined	(1)
CASIMO1	ncORF (SMIM22 locu ncORF (designated SMIM22)	Protein detected by MS	Not reported	Partial: siRNA depletion reduces proliferation; interacts with SQLE; evidence from gene depletion, not a somatic variant	Not reported	Breast	Not determined	(8)
33 shared lncRNA antigens (liver)	lncRNA-encoded	17 of 33 loci have Ribo-Seq translation support; remaining 16 have RNA prevalence (>10% of patients) but no direct translation evidence	13 of 33 loci have direct HLA peptide elution + LC-MS/MS confirmation. The remaining 20 are prevalence candidates (RNA-based) without confirmed immunopeptidomics evidence. Prevalence >10% is a population average across HLA allotype diversity.	Not individually tested for essentiality	Not reported for any of the 33; thymic absence inferred from RNA-seq data for the mRNA-prevalent subset; direct functional CD8^+^^+^; T cell recognition not demonstrated	Hepatocellular carcinoma	Various; allele-specific data not disaggregated	(6)
Pancreatic cryptic antigens	Various ncORFs	Yes: MS detection in patient-derived tumour material	Yes: HLA elution + LC-MS/MS from patient tumours	Not tested for essentiality	Yes: CD8^+^; T cell activation, cytokine production, cytotoxicity confirmed *in vitro*	Pancreatic	Various (patient-specific HLA)	(9)

Denominator notes: The ‘~40%’ figure (Introduction) refers to the proportion of tumour-specific antigens attributable to microproteins in a liver cancer meta-cohort, not the proportion of all detected antigens (6). The ‘33 lncRNA antigens’ designates lncRNA loci with >10% RNA-level prevalence across that patient meta-cohort; of these, 17 had Ribo-Seq translation support, and 13 had direct HLA peptide elution and LC-MS/MS confirmation (6). Prevalence, translation support, and HLA presentation evidence are three distinct and non-overlapping categories applicable to different subsets of the 33, they must not be conflated. All three figures derive from the same study and patient population (6) and do not constitute independent corroboration of each other.

Cancer cells sustain chronic ER stress, oxidative damage, and nutrient deprivation (18). These stresses converge on eIF2α phosphorylation by PERK, PKR, GCN2, and HRI, weakening canonical cap-dependent translation and permitting ribosomal scanning past standard AUG codons (19, 20). The result is a translational reprogramming: a stress-induced shift in the ribosomal machinery that permits uORF, non-AUG, and cryptic lncRNA translation products to be synthesised from transcripts already present in the cell, without necessarily requiring a change in transcript abundance (T1) (20, 21). This is the primary mechanism of H1, translational amplification without transcriptional change, and it is important not to conflate this with transcriptional upregulation of ncORF-encoding loci. Starck et al. demonstrated directly that persistent uORF translation under the ISR generates MHC class I ligands (T1,T2) (22). Oncogenes such as MYC and RAS can promote non-canonical translational start site usage, diversifying the population of ncORF translation products (23), which may provide a complementary and secondary mechanism that is not a prerequisite for ncORF peptide presentation under H1. eIF3d-mediated non-canonical initiation, which increases in several human cancers, constitutes a further translational mechanism (T1) (24). Cellular stress simultaneously elevates defective ribosomal products (DRiPs), rapidly synthesised and rapidly degraded fragments that the proteasome preferentially converts into antigen-presentation substrates (T1,T2) (25). The tumour cell surface thus partially encodes the cancer cell’s translational stress state. Essentiality constrains one, but only one, of the tumour’s options for reducing immunological exposure. Several dozen ncORFs are required for cancer cell survival (T1, T3-function) (1, 4), and these proteins must be translated; their constitutive turnover, misfolding, and quality-control degradation generate a peptide pool that can enter the MHC class I pathway via TAP transport and ERAP1 trimming (T2) (26, 27). Essentiality therefore constrains antigen loss through source-protein silencing by imposing a proliferative fitness cost. However, as established elsewhere in the field, only a small fraction of detectably translated proteins contributes to the MHC-I immunopeptidome (7); translation of an essential ncORF is necessary but not sufficient for surface presentation of any given epitope. Moreover, immune evasion via HLA class I downregulation, a well-documented tumour escape mechanism, would eliminate the peptide-HLA target despite continued protein expression and without any fitness cost from protein loss (35). H2’s essentiality constraint should therefore be understood as a conditional property that narrows the tumour’s escape options, not as a guarantee of antigen presentation. Silencing an essential ncORF may result in a fitness cost for cancer cells, potentially creating a presentation liability.

When these peptides reach the cell surface, the immune system may respond. Central tolerance operates through AIRE-controlled presentation of self-antigens on mTECs in the thymus; T cells bearing receptors that bind these presented self-peptides with sufficient affinity are deleted. However, it is important to establish the correct scope of this argument: ncORF-derived peptides are not absent from the thymic peptidome as a class. Existing large-scale datasets, including the HLA Ligand Atlas (40) and the Peptides for Cancer Immunotherapy (PCI) database (41), demonstrate that ncORF-derived peptides are detectable in healthy thymus and multiple other normal tissues. H3 therefore applies not to ncORFs in general, but specifically to the subset of cancer-associated lncRNA-encoded loci that are epigenetically silenced in mTECs under normal developmental conditions but are derepressed in tumour cells. Evidence for this specific subset is provided by Camarena et al., who found that 33 lncRNA-encoded antigens shared across more than 10% of liver cancer patients showed virtually no detectable expression in normal thymic tissue examined (6). For these specific loci, circulating T cell clones reactive to the encoded peptides may have been retained by avoiding negative selection. Two further distinctions are essential. First, absence of thymic mRNA expression is not equivalent to absence of peptide-HLA presentation on mTECs: mTECs can present peptides derived from transcripts below standard RNA-seq detection thresholds, and peptide abundance and transcript abundance are not linearly coupled. Prospective mTEC-specific immunopeptidomics using targeted LC-MS/MS with synthetic isotope-labelled peptide standards is required before thymic absence of any specific antigen can be confidently asserted. Second, and critically, thymic absence and peripheral safety are distinct: even if a given ncORF antigen is genuinely absent from the mTEC immunopeptidome, the corresponding T cells may cause off-tumour injury in healthy tissues that transiently present the same antigen under physiological stress (elaborated in Section 4.3). These two safety questions must be addressed independently and not conflated. The prominence of specific cancer-associated ncORF antigens in the immunopeptidome may reflect, in part, immune visibility shaped by an evolutionary developmental programme that keeps certain lncRNA loci transcriptionally silent in thymic tissue, a form of downward causation from the organism’s immune developmental history shaping T cell repertoire composition, but this remains a hypothesis awaiting prospective confirmation. The antigen-processing machinery provides a fourth potential source of enrichment, but with an important mechanistic clarification. The proteasome, TAP, ERAP1, and HLA form a selective filter, not a passive conduit. Short, disordered ncORF proteins with rapid turnover kinetics may be kinetically favoured substrates for DRiP-mediated proteasomal processing (T1,T2) (26, 27). ERAP1 trimming shapes the final peptide length distribution available for HLA loading; its loss shifts the immunopeptidome toward longer peptides (28), consistent with ERAP1 being a major determinant of length selection. The proposed enrichment in H4 operates at these upstream processing steps, proteasomal cleavage and ERAP1 trimming, where the structural and biochemical properties of ncORF source proteins (short length, disorder, elevated turnover) may favour production of HLA-compatible peptide lengths. Once a peptide is produced, imported into the ER, and presented for HLA loading, the peptide’s sequence and its compatibility with the HLA allele’s binding groove determine the biochemistry of peptide-HLA association identically for canonical and non-canonical sources. HLA-binding affinity is agnostic to cellular peptide origin; ncORF-derived peptides are not intrinsically ‘better binders’, and claims of more favourable anchor-residue profiles for ncORF peptides as a class are not currently supported by direct evidence. A further confound must be explicitly acknowledged: immunopeptidomics workflows detect short peptides more readily than tryptic proteomics, which may create a discovery bias that inflates the apparent representation of ncORF-derived sequences in the immunopeptidome relative to canonical proteins. Distinguishing genuine biological enrichment from this detection artefact requires matched canonical comparators of equivalent size, HLA-allele-stratified analysis, and normalisation against source protein translation rates and turnover, none of which has been systematically applied to ncORF sequences to date.

These mechanisms may compound one another (**Figure 1**). Oncogenic stress can expand the pool of cryptic translation products (H1); essentiality may constrain antigen loss through protein silencing for a subset (H2), though HLA downregulation remains an available evasion route; the tolerance gap may allow the immune system to respond when specific cancer-associated lncRNA antigens are presented (H3); and processing biases might enrich ncORF-derived sequences over their canonical competitors at the upstream processing level, not at the HLA-binding step (H4). This convergence, in which translational reprogramming, proliferative necessity, evolutionary immune history, and processing architecture may intersect at the microprotein level, provides a rationale to investigate whether ‘shared ncORF-derived peptide antigens’ are suitable candidate therapeutic targets when multiple hypotheses are simultaneously satisfied.

## 3 Toward clinical translation

The four-hypothesis framework may have important implications for target selection. Each causal mechanism defines both a potential vulnerability and a limitation, and distinguishing between them is crucial for identifying therapeutic targets that remain exposed to immune attack over time, rather than those that may be lost or downregulated during tumour evolution.

BioNTech, Enara Bio, and other groups are already constructing platforms to exploit shared non-canonical cancer antigens (29, 30), but target prioritisation remains largely empirical. The framework proposed here offers a conceptual mechanistic alternative for target selection. An antigen candidate that meets multiple criteria, subject to stress-induced translational reprogramming (H1), encoded by an essential locus (H2), with its specific lncRNA source locus epigenetically silenced in mTECs but derepressed in cancer (H3), and with source-protein properties that favour upstream antigen-processing steps (H4), is a more robust candidate than one supported by only a single criterion. Of these essentiality criteria are particularly actionable: if a tumour attempts to evade immune detection by silencing an essential ncORF, it would incur a proliferative fitness cost. This trade-off may help limit antigenic loss and maintain target exposure under immune selection pressure, unlike the escape dynamics often seen with conventional neoantigen vaccines in low-mutation tumours (9). Importantly, the co-satisfaction requirement of these criteria also addresses safety concerns. Because stress-induced translation under H1 is not exclusive to tumours, the integrated stress response (ISR) can activate in post-ischaemic tissues, virally infected cells, and chemotherapy-exposed normal cells, raising the risk that T cells primed against H1-driven antigens could also target stressed normal tissues. Therefore, for H1 candidates, it is preferable to select antigens whose encoding loci are also epigenetically silenced in normal tissues (H3), thereby reducing the risk of off-tumour effects, as discussed further in Section 4.3.

The central tolerance gap (H3) defines both the immunological opportunity and the primary safety unknown. If ncORF microproteins are genuinely absent from the thymic peptidome for specific cancer-associated lncRNA loci, the T cell repertoire should harbour reactive clones at relatively high precursor frequency. However, absence inferred retrospectively from tumour immunopeptidomics data (6) is not the same as prospectively confirmed absence in healthy-donor medullary thymic epithelial cells. This distinction is clinically critical: a vaccine inadvertently targeting a low-level thymic antigen risks autoimmunity. Resolving it, through mTEC-specific immunopeptidomics with targeted PRM methods in healthy donors, is one of the highest-priority experiments the field can perform.

Four technical gaps must close in parallel. First, prediction tools for antigen processing, not HLA binding itself, must be refined to account for the properties of ncORF source proteins. This distinction is critical: HLA-binding prediction algorithms assess whether a given peptide sequence is compatible with a specific HLA allele’s binding groove, and these algorithms are entirely agnostic to whether the peptide came from a canonical or non-canonical source. A peptide derived from an ncORF with the appropriate anchor residues will receive the same binding score as a canonical peptide with the same sequence; retraining HLA-binding algorithms on ncORF sequences would not improve binding predictions and is not warranted. The gap lies instead in predicting which peptides are produced from ncORF precursors, the upstream steps of proteasomal cleavage and ERAP1 trimming, and in models of these processing steps that could be refined with ncORF-specific features such as precursor length, structural disorder, and turnover rate. Separately, variant-effect predictors for assessing the functional impact of somatic mutations in microproteins do require retraining: tools such as SIFT (12), PolyPhen-2 (13), and AlphaMissense (14) were calibrated on evolutionarily conserved canonical proteins and lack the training data to reliably score variants in short, often disordered microproteins, where the sequence-function landscape is fundamentally different; microprotein deep mutational scanning datasets are needed for this purpose. Second, the distinction between T2 and T3 evidence must be enforced: immunopeptidomics detection (T2) is necessary but not sufficient; clinical utility requires demonstrated T cell recognition *in vivo* (T3) (9, 15), and the neoantigen vaccine literature shows these do not reliably coincide. Third, immunopeptidomics workflows must incorporate non-canonical ORF sequence databases with rigorous false discovery rate control, including group FDR approaches to account for the expanded search space; this practice is now increasingly adopted as standard in the field but is not yet universal (2, 15–17); without it, the most clinically relevant antigens remain invisible to discovery pipelines. Fourth, prospective thymic immunopeptidomics in healthy donors must employ targeted LC-MS/MS methods, specifically parallel reaction monitoring (PRM) with synthetic isotope-labelled peptide standards, for the specific candidate antigens under evaluation, rather than data-dependent acquisition alone. Although resources such as the HLA Ligand Atlas (40) and PCI database (41) provide an invaluable baseline for established antigens, the sensitivity of untargeted data-dependent methods is insufficient to exclude low-level presentation of novel ncORF-derived peptides that could nonetheless drive tolerance or off-tumour toxicity for specific high-priority candidates. The next generation of cancer vaccines may include targets derived from previously unannotated, non-canonical regions of the genome, sometimes referred to as the ‘dark genome’, in addition to those from somatic mutations in canonical genes. Developing such therapies may benefit from viewing microproteins as structurally constrained, potentially functionally important, and immunologically visible molecular features of cancer biology (**Figure 1**), which may be targetable due to their roles in cancer cell survival.

Beyond T cell-based immunotherapy, the ncORF-derived peptides presented on HLA class I offer additional routes for therapeutic engagement that do not depend on the T cell tolerance gap. TCR mimetic antibodies, which recognise specific peptide-HLA complexes as their target antigens and direct immune effector functions against antigen-positive cells without requiring endogenous T cell involvement, are an emerging modality that could exploit ncORF-derived tumour surface antigens while bypassing constraints imposed by central tolerance (42). *De novo* designed proteins with high affinity and selectivity for defined pMHC complexes, engineered using computational protein design, have been recently described; these binders facilitate T cell-mediated cytotoxicity against antigen-positive cancer cells (43) by engaging pMHC-displaying tumour cells in a manner that can be independent of endogenous TCR priming. These approaches expand the therapeutic landscape for ncORF antigens beyond the subset satisfying the H3 tolerance gap criterion, and they merit evaluation alongside T cell vaccine strategies in any platform assessing this antigen class. For essential ncORF antigens (H2) where thymic tolerance may be partially intact, antibody-based pMHC targeting could be effective where a T cell vaccine would risk autoimmunity.

## 4 Discussion

The preceding sections have presented multi-level evidence suggesting that ncORF-derived microproteins populate the cancer immunopeptidome through several biological mechanisms, and that their translation into therapeutic targets will require new computational tools, more rigorous immunopeptidomics workflows, and prospective thymic peptidome characterisation. It is now important to consider how this body of evidence fits within the broader history of shared-antigen oncology, clarify the unique mechanistic contributions of this approach, identify limitations of the current evidence base that have not yet been discussed, and define the evidential standard required for proof-of-concept.

### 4.1 A distinct route to immunological visibility

The ambition to exploit shared, non-mutational antigens for cancer immunotherapy is not new. Cancer-testis antigens were proposed as vaccine targets over three decades ago: their restricted normal-tissue expression, epigenetic silencing in somatic cells, and recurrence across patient populations appeared to satisfy the key criteria for shared-antigen design (31, 32). Clinical outcomes have been largely disappointing, and the reasons are instructive (32). Evidence from studies of endogenous retroelements, and by analogy CT antigens, suggests that tolerance induction is incomplete when encoding sequences show even low-level expression in thymic antigen-presenting cells (33); intratumoral expression is frequently heterogeneous; and functional importance in tumour biology, a property that might constrain immune evasion, has rarely been demonstrated (30). Retroelement sequences, a class recently explored, share the epigenetic derepression logic and largely avoid tolerance induction. These events are driven by global epigenetic erosion rather than cancer-cell requirements, and they cannot be expected to resist antigenic loss under immune pressure (34, 35).

The ncORF framework proposed in this article occupies conceptually distinct ground, though with important caveats about class boundaries. Transposable elements (TEs) are among the genomic sources of ncORF-encoded translation products; retroelement-derived sequences and ncORF-encoded microproteins therefore overlap and are not mutually exclusive categories. The boundaries between ncORF-derived, retroelement-derived, and cancer-testis antigen classes are permeable, and a rigorous antigen classification framework should account for these overlaps rather than treating them as discrete sets. Furthermore, essentiality as a constraint on antigenic loss is a conceptual property that could, in principle, apply to any antigen class, including cancer-testis antigens or retroelement products, if a given member of those classes were shown to be functionally essential for cancer cell survival. The selective advantage proposed here does not arise from any intrinsic class-wide property of ncORFs; it arises from having a systematic multi-hypothesis framework to identify and prioritise candidates that simultaneously satisfy essentiality (H2), a cancer-specific tolerance gap (H3), stress-induced translational amplification (H1), and antigen-processing enrichment (H4), a combination that no individual antigen class can be assumed to satisfy at the population level. The tolerance gap, where it is confirmed prospectively in mTECs, arises from a developmental epigenetic programme that silences specific lncRNA loci in thymic tissue while permitting cancer-driven derepression in somatic cells, mechanistically distinct from the germline immune privilege of cancer-testis antigens and the phylogenetic novelty of retroelements, but not representing a categorically superior property. The defensible and novel contribution of this framework is its combination of selection criteria as a prioritisation tool, not an established class-wide superiority. The empirical question of whether essentiality and a *bona fide* thymic tolerance gap co-occur at the same ncORF loci, rather than characterising disjoint subsets, has not yet been directly addressed in the literature and remains a priority for the field.

### 4.2 Immune editing in the presence of an essentiality constraint

Classical cancer immunoediting predicts progressive attrition of immunological targets: immune recognition selects for antigen-loss variants or for tumours that have downregulated HLA class I surface expression, ultimately eroding the detectable immunopeptidome (35). For non-essential ncORF antigens driven predominantly by stress-induced translation (H1), this erosion is plausible: tumour cells could reduce antigen presentation by moderating translational stress through metabolic adaptation, or by selecting for epigenetic silencing of non-essential ncORF loci under immune pressure. For essential ncORF antigens, the prediction is different. Antigen loss through ncORF silencing should be constrained by the proliferative cost of losing the microprotein’s function, leaving the tumour with the alternatives of HLA downregulation, induction of T cell exhaustion, and microenvironmental immunosuppression, routes that do not remove the antigen but restrict immune effector access.

It is important to note that HLA downregulation as a response to immune pressure is not merely a restriction on effector access: complete HLA class I loss would eliminate the peptide-HLA target entirely despite the continued presence of the essential source protein, effectively circumventing H2’s essentiality constraint by an antigen-display mechanism rather than an antigen-source mechanism. Longitudinal immunopeptidomics of serially biopsied tumours in clinical trials should therefore monitor for both antigen-loss (source-protein silencing) and HLA-loss (display-level silencing) escape events as distinct readouts. This difference is testable: under sustained antigen-specific immune pressure *in vivo*, through vaccination or adoptive cell transfer, essential ncORF antigens should exhibit lower rates of antigen-loss escape than non-essential comparators. Incorporating longitudinal immunopeptidomics of serially biopsied tumours into early clinical trials, specifically designed to detect antigen-loss versus HLA-loss escape, would generate data of direct mechanistic and strategic value. No currently published study has been designed to address this distinction for ncORF-derived antigens.

### 4.3 A safety dimension not yet accounted for

The clinical translation section correctly identifies the risk of targeting an ncORF antigen with residual thymic expression as the primary safety concern for H3-driven candidates. A separate safety consideration applies specifically to antigens driven by H1, stress-induced non-canonical translation. Oncogenic stress is, by definition, chronic and qualitatively distinct from physiological stress responses, but the integrated stress response itself is not tumour-exclusive. Post-ischaemic tissues, virally infected cells, transplanted organs undergoing rejection, and cells exposed to cytotoxic chemotherapy all activate eIF2α phosphorylation and may transiently generate overlapping non-canonical translation products (36). A T cell response primed against an H1-driven ncORF antigen during vaccination could theoretically recognise transiently presenting stressed normal tissues during episodes of systemic physiological stress that occur concomitantly with treatment. This risk is not a contraindication, but it argues for a specific selection criterion: H1-candidate antigens should be preferentially chosen where stress-induced translation is amplified by cancer-specific epigenetic derepression of the encoding locus, effectively requiring co-satisfaction of H1 and H3, and inflammatory toxicity should be monitored with particular attention during cycles of cytotoxic therapy in clinical trials.

The distinction between thymic absence and peripheral off-tumour risk elaborated here reinforces the point made in the H3 framework: these are two independent safety questions requiring independent experimental answers. A candidate antigen may clear the thymic safety threshold (confirmed absent from the mTEC immunopeptidome by targeted PRM) while still presenting risk in stressed peripheral normal tissues, and vice versa. Clinical monitoring protocols in early-phase trials should be designed to detect both forms of off-tumour toxicity as distinct signals.

### 4.4 Discordant screens, cross-sectional evidence, and the population HLA question

The discordance between the essentiality results of Prensner et al. (4) and Deutsch et al. (1) on one hand and Schlesinger et al. (5) on the other, briefly noted in the Introduction, deserves more direct methodological engagement. The variability is not surprising given that CRISPR viability screens are sensitive to guide RNA library composition, cell line metabolic state, screen duration, and the distinction between acute loss-of-function effects and chronic fitness consequences (37). What it implies is that individual screens, however large, should not be treated as definitive catalogues. Pre-registered meta-analyses of harmonised CRISPR screen datasets, applying explicit statistical models for effect-size heterogeneity and guide-library composition effects, are needed before any ncORF locus is progressed as a validated essential target for clinical development. The same concern applies more broadly to the field’s evidentiary base: most immunopeptidomics studies are cross-sectional snapshots. They describe which ncORF peptides are presented at a given tumour stage, under prevailing oncogenic stress and epigenetic conditions, but provide no information about whether that presentation is stable over the course of disease progression, shifts under therapeutic pressure, or varies with the metabolic and immune state of the tumour microenvironment at different treatment timepoints. Longitudinal cohorts with matched pre-treatment and on-treatment tumour sampling are needed before assessing the therapeutic durability of ncORF antigen presentation responsibly.

A further complication for shared-antigen design deserves recognition. Prevalence statistics, such as the occurrence of specific lncRNA-encoded antigens in more than 10% of liver cancer patients, are population averages that aggregate across HLA allotype diversity. Since HLA class I alleles vary substantially in their peptide-binding preferences, a given ncORF-derived peptide may be on the surface of tumours in 10% of the overall patient population while being absent from the immunopeptidome of patients carrying certain HLA supertypes. Population-scale HLA coverage modelling, analogous to that routinely applied in infectious disease vaccine design, will be necessary to determine the actual proportion of patients in any target indication for whom a shared ncORF vaccine is likely to generate a surface antigen and thus a productive T cell response (38, 39). Without this analysis, prevalence figures should be treated as upper-bound estimates of therapeutic reach rather than direct measures of vaccine applicability.

### 4.5 What proof-of-concept may require

The field has accumulated Tier 1 and Tier 2 evidence across multiple tumour types, and the first Tier 3 demonstrations, direct CD8^+^; T cell recognition of ncORF-derived cryptic antigens, are beginning to emerge. The next threshold, however, may not be more detection but mechanistic closure: a demonstration that a multi-hypothesis-satisfying ncORF antigen drives a protective and durable immune response *in vivo*, that this response is constrained from antigen-loss escape by the essentiality-fitness trade-off, and that no comparable antigen presentation occurs in stressed normal tissues at biologically relevant levels. To satisfy this standard will require coordinated experimental programmes spanning: (1) mTEC-specific immunopeptidomics in healthy donors using targeted PRM with validated synthetic peptide standards for the specific antigens under evaluation; (2) patient-derived organoid-based or isogenic cell-line-based essentiality validation under sustained immune pressure, measuring both antigen-loss (source-protein silencing) and HLA-loss (display-level) escape rates separately; and (3) early-phase clinical monitoring protocols capable of detecting off-tumour inflammatory toxicity in both non-stressed and physiologically stressed normal tissues, not only in the primary tumour bed. The standards established for neoantigen vaccine evaluation, peptide-MHC tetramer frequency, functional cytotoxicity assays, and tumour regression endpoints apply here, but with the essential additions of longitudinal antigen stability monitoring as a primary readout and prospective documentation of the escape route used (antigen-loss versus HLA-loss) when responses are not durable.

The innovative framework in this article suggests that a cancer cell’s immunological identity might not be reduced to its somatic mutation inventory. Translational biochemistry, cellular stress physiology, evolutionary immune history, and the structural selectivity of antigen processing all shape the surface the immune system encounters, independently and in combination. The ncORF antigen class may represent a concrete manifestation of this principle, and its translational potential will likely be realised only if the field adopts discovery and validation pipelines explicitly calibrated to the biological complexity described here, integrating multi-tier evidence, longitudinal tumour monitoring, prospective thymic safety characterisation, and targeted MS methods, rather than those inherited unchanged from the canonical neoantigen era. Concise methodological frameworks for integrating multi-omics data streams in candidate prioritisation and for structuring translational validation programmes have been described and provide practical guidance for implementing these pipelines (44, 45).

## Glossary

Non-canonical open reading frame (ncORF): A continuous sequence of typically fewer than 100 codons long uninterrupted by stop codons, sometimes defined to start with ATG/AUG
Short ORF (sORF): An open reading frame of typically fewer than 100 codons long, defined by length rather than genomic context. sORFs and ncORFs overlap substantially but are not interchangeable: sORFs can occur within canonical protein-coding mRNAs (as upstream ORFs, uORFs) as well as within non-coding regions, while ncORFs are defined by their non-canonical genomic location regardless of length. The Schlesinger et{nb/}al. screen (5) employs ‘sORF’ to emphasise non-canonical genomic origin. When these terms appear in different cited studies, direct comparison of their catalogues requires accounting for these definitional differences
Peptidein: A translation product detected from an ncORF whose biological function has not yet been defined; a provisional annotation introduced by the TransCODE consortium to distinguish detected peptides from functionally characterised proteins
Microprotein: A small functionally characterised protein, with evolutionary selection or experimental evidence of presumed functionality, typically fewer than 100 amino acids, often encoded by an ncORF and frequently intrinsically disordered
Immunopeptidome: The complete set of peptide antigens displayed on the cell surface by human leukocyte antigen (HLA) class I (or class II) molecules; it represents the antigenic face of a cell visible to the immune system
HLA immunopeptidomics: A mass spectrometry approach in which HLA molecules are immunoprecipitated from cells, the bound peptides are eluted, and their sequences are identified by liquid chromatography tandem mass spectrometry (LC-MS/MS)
Defective ribosomal products (DRiPs): Newly synthesised proteins that fail to fold correctly and are degraded within minutes of translation; they are a major source of substrates for HLA class I antigen presentation
Integrated stress response (ISR): A conserved cellular signalling network triggered by stresses such as nutrient deprivation, hypoxia, or ER stress; it phosphorylates eIF2α and reprograms translation initiation to favour non-canonical start sites
Central tolerance: The process occurring in the thymus during T cell development, in which T cell receptors that bind strongly to self-peptides presented by HLA molecules trigger deletion, thereby preventing autoimmunity
Bidirectional causation: The principle or concept that biological causation flows both upward (from genes and molecules to cells and organisms) and downward (from the organism and its environment to molecular processes), with no single level holding privileged explanatory authority
Pan-essentiality: A property of a gene or ORF whose inactivation impairs survival across a broad spectrum of cell lines or cancer types, indicating a non-redundant function in cellular fitness
ERAP1: Endoplasmic reticulum aminopeptidase 1; an enzyme that trims N-terminal residues from precursor peptides inside the ER, generating peptides of optimal length for stable binding to HLA class I molecules
TAP: Transporter associated with Antigen Processing; a heterodimeric ATP-binding cassette transporter located in the endoplasmic reticulum membrane that actively imports proteasome-generated peptides from the cytosol into the ER lumen for loading onto nascent MHC class I molecules
Ribo-seq: Ribosome profiling; a deep sequencing technique that maps ribosome-protected mRNA fragments genome-wide, providing nucleotide-resolution evidence of active translation, including at non-canonical start sites
Deep mutational scanning: A high-throughput experimental method in which libraries of thousands to millions of protein variants are generated and simultaneously assayed for functional consequences, enabling systematic mapping of sequence-fitness relationships
CRISPR: Clustered Regularly Interspaced Short Palindromic Repeats; an adaptive immune system from bacteria repurposed as a programmable genome-editing tool, commonly used to introduce targeted gene knockouts or activation/repression to assess gene essentiality
MHC class I: Major Histocompatibility Complex class I; a heterodimeric cell-surface glycoprotein that binds endogenous peptides and presents them to CD8+ cytotoxic T lymphocytes, serving as the primary surveillance mechanism for intracellular protein content
Medullary thymic epithelial cell (mTEC): A specialised epithelial cell type residing in the thymic medulla that expresses the autoimmune regulator (AIRE) and presents tissue-restricted antigens to developing T cells, thereby enforcing central tolerance through negative selection
ER stress: Endoplasmic reticulum stress; a cellular condition arising from accumulation of misfolded proteins or metabolic imbalance in the ER lumen, which activates the unfolded protein response and can reprogram global translation via eIF2α phosphorylation
eIF3d-mediated non-canonical initiation: A mode of translation initiation in which the eIF3d subunit of the eukaryotic initiation factor 3 complex binds directly to specific mRNA structural elements, recruiting the 40S ribosomal subunit independently of the canonical 5′ cap-binding protein eIF4E
Evolutionary Trap: The convergence of H2 (essentiality) and H3 (tolerance gap) at the same ncORF locus, such that the tumour cannot silence the antigen source without incurring a proliferative fitness cost (H2) while the immune system retains the capacity to recognise the presented peptide as foreign (H3). This integration is proposed as the basis for a therapeutically durable target, one resistant to antigen-loss escape, and is distinct from either property alone.
